# A Robust Interacting Multi-Model Multi-Bernoulli Mixture Filter for Maneuvering Multitarget Tracking under Glint Noise

**DOI:** 10.3390/s24092720

**Published:** 2024-04-24

**Authors:** Benru Yu, Hong Gu, Weimin Su

**Affiliations:** School of Electronic and Optical Engineering, Nanjing University of Science and Technology, Nanjing 210094, China; benruyu@njust.edu.cn (B.Y.); suweimin@mail.njust.edu.cn (W.S.)

**Keywords:** variational Bayesian, multivariate Laplace distribution, glint noise, multi-Bernoulli mixture filter, interacting multi-model algorithm, maneuvering target tracking

## Abstract

In practical radar systems, changes in the target aspect toward the radar will result in glint noise disturbances in the measurement data. The glint noise has heavy-tailed characteristics and cannot be perfectly modeled by the Gaussian distribution widely used in conventional tracking algorithms. In this article, we investigate the challenging problem of tracking a time-varying number of maneuvering targets in the context of glint noise with unknown statistics. By assuming a set of models for the possible motion modes of each single-target hypothesis and leveraging the multivariate Laplace distribution to model measurement noise, we propose a robust interacting multi-model multi-Bernoulli mixture filter based on the variational Bayesian method. Within this filter, the unknown noise statistics are adaptively learned while filtering and the predictive likelihood is approximately calculated by means of the variational lower bound. The effectiveness and superiority of our proposed filter is verified via computer simulations.

## 1. Introduction

The aim of multitarget tracking (MTT) lies in estimating the kinematic state (e.g., position, velocity, acceleration) of each target within the region under surveillance from measurement data provided by sensing devices (e.g., radar, sonar, microphone) [1]. Often, the number of targets may change over time as a result of the stochastic births, deaths, and spawns of targets. Moreover, merely part of the available measurements originates from targets, and the association maps between targets and measurements are not clear. The random finite set (RFS) [2] offers a natural formulation of the multitarget states and multitarget measurements. Using RFS modeling, the probability hypothesis density (PHD) filter [3], cardinalized PHD (CPHD) filter [4], and multi-Bernoulli filters [2,5] were developed as tractable MTT algorithms. Recently, driven by the development of multitarget conjugate priors, the advanced generalized labeled multi-Bernoulli (GLMB) filter [6], multi-Bernoulli mixture (MBM) filter [7], and Poisson MBM (PMBM) filter [8] were reported by researchers, which admit closed-form Bayesian recursion without certain approximations necessary in the PHD, CPHD, and multi-Bernoulli filters. By approximating a GLMB with a single term, an efficient alternative to the GLMB filter named the labeled multi-Bernoulli (LMB) filter [9] was proposed to make compromise between complexity and accuracy. Performance evaluations of the GLMB, PMBM, MBM, and LMB filters can be found in [10].

Most MTT algorithms assume that all targets follow the same dynamic model throughout. However, this assumption is too ideal in practice. For example, in a battlefield environment, a fighter jet has to carry out a series of tactical maneuvers to avoid being locked on by enemy weapon systems. In this case, these algorithms demonstrate poor performance. The jump Markov system or multi-model (MM) approach [11] has proven to be effective for maneuvering target tracking, in which the target can switch among a set of dynamic models in a Markovian fashion. By applying the MM approach in conjunction with RFS-based multitarget filters, numerous maneuvering MTT (MMTT) algorithms have been reported in [12,13,14,15,16,17,18,19,20]. A fundamental premise of these algorithms is that the measurement noise is Gaussian distributed with known statistics. However, this is not necessarily the case in practical radar systems, where changes in the target aspect with respect to the radar can cause the apparent center of radar reflections to wander significantly. The random wandering of the apparent radar reflecting center dramatically increases the radar cross-section fluctuations [21,22,23,24,25], resulting in significant glint noise. It was found that the glint noise has a heavy-tailed probability density function (PDF) and cannot be perfectly modeled by the Gaussian distribution. In general, a priori knowledge of the glint noise statistics is not available.

The common approach for modeling glint noise is to exploit a type of distribution or the combination of multiple distributions. In [26], the Student’s *t* (ST) distribution was used to model glint noise, whereas the mixture of Gaussian distributions was used in [27]. In [28], the glint noise was modeled by the mixture of a Gaussian distribution and a Laplacian distribution. In particular, the ST distribution is immune to measurement outliers and has been widely used in RFS-based MTT [29,30,31], which can accurately characterize the tailed behavior by carefully selecting its degree of freedom (DOF) parameter. Based on the ST distribution and variational Bayesian (VB) method [32], a robust MMTT algorithm was proposed under the marginal distribution Bayes (MDB) filtering framework [33]. The resulting ST-MM-MDB filter can adaptively learn the unknown scale matrix and DOF parameter of the ST distribution while filtering. In addition, the ST-MM-LMB filter was also reported in [34], which adopts a similar idea to estimate unknown noise statistics. However, as indicated in [35], these ST-based filters cannot estimate the DOF parameter accurately by means of limited measurement samples. To mitigate this drawback, attempts have been made to model glint noise using the multivariate Laplace (ML) distribution [36], which avoids the selection of the DOF parameter. A related Kalman filter was presented in [37], which shows higher estimation accuracy than the existing ST-based counterpart [38]. Extensions and improvements of this filter were reported in [39,40]. These works, however, are limited to the case where there is only a single non-maneuvering target to be tracked using one noisy measurement per scan. When multiple maneuvering targets are involved, a series of complicated factors, e.g., the stochastic appearances and disappearances of targets, unknown target–measurement association, misdetection (a target is detected by radar with a certain probability), and false alarms (spurious measurements not originating from any target) need to be systematically considered. It is infeasible to extend the single non-maneuvering case to the maneuvering multitarget case straightforwardly.

Motivated by the above discussions, in this article, we propose a robust MMTT algorithm in the context of glint measurement noise based on the MBM filter and ML distribution. Specifically, we assume a set of models for each Bernoulli component (BC) in the MBM representing a single-target hypothesis so as to capture the real-time maneuvers of targets. Moreover, we employed the ML distribution to model measurement noise and formulate the corresponding likelihood function as a Gaussian scale mixture form, leading to a hierarchical measurement model. Based on the MM assumption and such a hierarchical model, we derive a robust MBM filter for maneuvering targets using strategies from the interacting MM (IMM) algorithm (a celebrated MM approach with high cost effectiveness) [41], in which the unknown noise statistics are adaptively estimated by means of the VB method accompanied by IMM-MBM filtering. Since the involved predictive likelihood cannot be exactly calculated, we make use of the variational lower bound to obtain an approximate alternative. To summarize, the main contributions of this article are as follows:

(1) Based on ML modeling and the VB method, a robust IMM-MBM filter is proposed to adaptively learn unknown glint noise statistics while filtering.
(2) A series of numerical simulations is performed to test the robustness of the proposed algorithm and compare its performance with the existing solutions.

The remainder of this article is organized as follows. Section 2 provides the necessary background knowledge. Section 3 reviews the standard (single-model) MBM filter and its Gaussian implementation. Section 4 details the robust MMTT algorithm proposed in this article. Section 5 presents the simulation results. Section 6 gives closing remarks.

## 2. Background

This section gives the notations used throughout this article and reviews several kinds of RFSs relevant to the development of our key results, as well as the ST and ML distributions.

### 2.1. Notation

In this article, tr(·) and det(·) denote the trace and determinant of a matrix, respectively; E[·] denotes the expectation operator; N(·;m,P) denotes the Gaussian PDF with mean vector m and covariance matrix P; IW(·;Ψ,ν) denotes the inverse Wishart (IW) PDF with scale matrix Ψ and DOF parameter ν; GIG(·;a,b,c) denotes the generalized inverse Gaussian (GIG) PDF with shape parameters *a*, *b*, and *c*; E(·;λ) denotes the exponential PDF with rate parameter λ; the superscripts −1 and ⊤ denote the inverse operation and transpose operation, respectively; exp denotes the natural exponential; log denotes the natural logarithm.

### 2.2. RFS Statistics

An RFS is essentially a set-valued random variable of which both the cardinality (number of elements) and the elements are random. Akin to conventional random variables, the randomness of an RFS is entirely described by its probability density. Several kinds of RFSs of interest are given next.

A Poisson RFS X is an RFS with the cardinality $\left|X\right|$ being Poisson distributed with mean $\bar{N}=\int D\left(x\right)dx$, where D(x) denotes the intensity, also known as the PHD, and the elements x∈X are independent and identically distributed in light of the spatial density $D\left(x\right)/\bar{N}$ for any finite cardinality. The probability density of the Poisson RFS X is given by [2] (pp. 366)

(1) $$\begin{array}{c} f^{p}\left(X\right)=\exp\left(-\int D(x)dx\right)\prod_{x\in X}D\left(x\right). \end{array}$$

Clearly, the Poisson RFS density $f^{p}\left(X\right)$ is entirely determined by the intensity D(x).

A Bernoulli RFS X can either be empty, i.e., X=∅, with probability 1−r, or be composed of a single element x, i.e., $X=\left\{x\right\}$, with probability *r*. Conditional on being nonempty, x is distributed according to the spatial density *p*. The probability density of the Bernoulli RFS X takes the form [2] (pp. 368)

(2) $$\begin{array}{c} f^{b}\left(X\right)=\left\{\begin{array}{cc} 1-r, & X=\emptyset ,\\ rp(x), & X=\left\{x\right\}. \end{array}\right. \end{array}$$

A multi-Bernoulli (MB) RFS X is a union of a fixed number of independent Bernoulli RFSs, i.e., $X={⊎}_{i\in I}X^{i}$, where ⊎ denotes the disjoint union, I is an index set for BCs in the MB RFS, and $X^{i}$ denotes the *i*-th BC. The probability density of the MB RFS X is given by [7]

(3) $$\begin{array}{c} f^{\mathrm{mb}}\left(X\right)=\sum_{{⊎}_{i\in I}X^{i}=X}\prod_{i\in I}f^{i}\left(X^{i}\right). \end{array}$$

An MBM RFS X is a normalized and weighted sum of MB RFSs, whose probability density is given by [7]

(4) $$\begin{array}{c} f^{\mathrm{mbm}}\left(X\right)\propto \sum_{j\in J}\sum_{{⊎}_{i\in I^{j}}X^{i}=X}\prod_{i\in I^{j}}w^{i,j}f^{i,j}\left(X^{i}\right), \end{array}$$

where ∝ stands for proportionality, J is the index set for MB components in the MBM, $I^{j}$ is the index set for BCs in the *j*-th MB RFS, and $w^{i,j}$ and $f^{i,j}\left(X^{i}\right)$ are the respective weight and probability density of the *i*-th BC in the *j*-th MB RFS.

### 2.3. ST and ML Distributions

The ST distribution is widely used to model glint noise. For a *d*-dimensional ST distributed random variable x, its PDF is given by [42]

(5) $$\begin{array}{c} f^{\mathrm{st}}\left(x\right)=\frac{\Gamma (\frac{\nu +2}{2})}{\Gamma \left(\frac{\nu }{2}\right){\left(\nu \pi \right)}^{\frac{d}{2}}\sqrt{\det(P)}}{\left(1+\frac{\Delta^{2}}{\nu }\right)}^{-\frac{\nu +2}{2}}, \end{array}$$

where Γ(·) denotes the Gamma function, ν denotes the DOF parameter, $\Delta^{2}={\left(x-m\right)}^{⊤}P^{-1}\left(x-m\right)$, m denotes the mean vector, and P denotes the scale matrix. Note that the covariance matrix of x is $\frac{\nu }{\nu -2}P$(ν>2) instead of P. The tailed behavior of the ST PDF $f^{\mathrm{st}}\left(x\right)$ is dominated by the DOF parameter ν. More specifically, the smaller the DOF parameter ν, the heavier the tail, and vice versa. When the DOF parameter ν tends to infinity, the ST PDF $f^{\mathrm{st}}\left(x\right)$ becomes a Gaussian PDF.

When exact statistics of glint noise are not available, robust ST-based Kalman filters [43,44] have been proposed to jointly estimate the state vector and unknown noise parameters. However, as indicated in [35], these filters cannot estimate the DOF parameter accurately by means of limited measurement samples. Such a drawback motivates the employment of the ML distribution to model glint noise, which gets rid of the intractable DOF parameter. For a *d*-dimensional ML distributed random variable x, its PDF is given by [45]

(6) $$\begin{array}{c} f^{\mathrm{ml}}\left(x\right)=\frac{2{\left(\sqrt{\frac{\varrho (x)}{2}}\right)}^{\frac{1}{2}\left(1-\frac{d}{2}\right)}}{{\left(2\pi \right)}^{\frac{d}{2}}\sqrt{\det(P)}}B_{\frac{d}{2}-1}\left(\sqrt{2\varrho (x)}\right), \end{array}$$

where $\varrho \left(x\right)={\left(x-m\right)}^{⊤}P^{-1}\left(x-m\right)$, m is the mean vector, P is the covariance matrix, and $B_{\nu }$ denotes the modified Bessel function of the second kind with order ν. As shown in [37], the ML PDF $f^{\mathrm{ml}}\left(x\right)$ can be reformulated as a Gaussian-scale mixture form:

(7) $$\begin{array}{cc} f(x|\zeta ) & =N(x;m,\zeta P), \end{array}$$

(8) $$\begin{array}{cc} f(\zeta ) & =E(\zeta ;\lambda ), \end{array}$$

where ζ is an auxiliary variable.

## 3. Gaussian MBM Filter

The MBM filter is closed to the Bayes prediction and update steps, in which the multitarget density at time *t*, $t\in \left\{k,k+1\right\}$, conditional on the measurements up to time *k* is an MBM density of the form [46]

(9) $$f_{t|k}\left(X_{t}\right)\propto \sum_{a\in A_{t|k}}\sum_{{⊎}_{l=1}^{n_{t|k}}X^{\ell }=X_{t}}\prod_{i=1}^{n_{t|k}}\left[w_{t|k}^{i,a^{i}}f_{t|k}^{i,a^{i}}\left(X^{i}\right)\right].$$

An explanation of expression (9) is given as follows. To begin with, $X_{t}$ is the multitarget state RFS at time *t*, $n_{t|k}$ is the number of BCs, and $a^{i}=\left(\tau^{i},\ell^{i},\chi_{\tau^{i}:k}^{i}\right)$ denotes the single-target hypothesis in regard to the *i*-th BC with $\tau^{i}$, $\ell^{i}$, and $\chi_{t^{i}:k}^{i}=\left(\chi_{\tau^{i}}^{i},\ldots ,\chi_{k}^{i}\right)$ being the birth time, birth index, and data associations up to time *k*, respectively. At a specific time *j*, $\chi_{j}^{i}=0$ if the *i*-th BC is misdetected and $\chi_{k}^{j}=p\in \left\{1,\cdots ,m_{k}\right\}$ if the *i*-th BC is associated with the *p*-th measurement, where $m_{k}$ is the number of measurements available at time *k*. Moreover, $a=\left(a^{1},\cdots ,a^{n_{t|k}}\right)$ denotes a global hypothesis, which is composed of all single-target hypotheses, and $A_{t|k}$ denotes the collection of all global hypotheses. The *i*-th BC with single-target hypothesis $a^{i}$ has an associated weight $w_{t|k}^{i,a^{i}}$ and probability density $f_{t|k}^{i,a^{i}}$ of the form (2) characterized by the existence probability $r_{t|k}^{i,a^{i}}$ and spatial density $p_{t|k}^{i,a^{i}}$. One recursion of the MBM filter is summarized below; see [7,8] for the mathematical proofs.

**Proposition** **1** (prediction). *Assume that the filtering density at time k is of the form of (9) with t=k. Then, the predicted density involves $n_{k+1|k}$ BCs, where $n_{k+1|k}=n_{k|k}+n_{k+1}^{b}$, and $n_{k|k}$ and $n_{k+1}^{b}$ are the number of BCs characterizing surviving targets and newborn targets, respectively. For each surviving BC $i\in \left\{1,\cdots ,n_{k|k}\right\}$, the predicted parameters are calculated by*

(10) $$\begin{array}{cc} w_{k+1|k}^{i,a^{i}} & =w_{k|k}^{i,a^{i}}, \end{array}$$

(11) $$\begin{array}{cc} r_{k+1|k}^{i,a^{i}} & =r_{k|k}^{i,a^{i}}\int p_{S,k}\left(x\right)p_{k|k}^{i,a^{i}}\left(x\right)dx, \end{array}$$

(12) $$\begin{array}{cc} p_{k+1|k}^{i,a^{i}}\left(x\right) & =\frac{\int p_{S,k}\left(x^{\prime }\right)p_{k|k}^{i,a^{i}}\left(x^{\prime }\right)\varphi_{k+1|k}\left(x|x^{\prime }\right)dx^{\prime }}{\int p_{S,k}\left(x^{\prime }\right)p_{k|k}^{i,a^{i}}\left(x^{\prime }\right)dx^{\prime }}, \end{array}$$

*where $p_{S,k}\left(x\right)$ and $\varphi_{k+1|k}\left(x|x^{\prime }\right)$ denote the probability of survival and single-target state transition density, respectively. For each newborn BC $i\in \left\{n_{k|k}+1,\cdots ,n_{k+1|k}\right\}$, we have*

(13) $$\begin{array}{cc} a^{i} & =(k+1,i-n_{k|k}), \end{array}$$

(14) $$\begin{array}{cc} w_{k+1|k}^{i,a^{i}} & =1, \end{array}$$

(15) $$\begin{array}{cc} r_{k+1|k}^{i,a^{i}} & =r_{k+1}^{b,i-n_{k|k}}, \end{array}$$

(16) $$\begin{array}{cc} p_{k+1|k}^{i,a^{i}}\left(x\right) & =p_{k+1}^{b,i-n_{k|k}}\left(x\right). \end{array}$$

*Here, the single-target hypothesis reduces to $a^{i}=\left(\tau^{i},\ell^{i}\right)$ since there has not been a data association event for newborn targets yet.*

**Proposition** **2** (update). *The number of BCs does not change in the update step, so $n_{k|k}=n_{k|k-1}$. For each BC $i\in \left\{1,\cdots ,n_{k|k}\right\}$ with single-target hypothesis $a^{i}$, it generates a misdetection hypothesis and one association hypothesis for each measurement $z_{k}^{j}$ in the current measurement set $Z_{k}=\left\{z_{k}^{1},\cdots ,z_{k}^{m_{k}}\right\}$. Note that $Z_{k}$ is composed of target-originated measurements and false alarms not originating from any target. To distinguish these two hypotheses, we make use of an ordered pair of integers $\left(a^{i},p\right)$ with $p\in \left\{0,\cdots ,m_{k}\right\}$. Under the misdetection hypothesis, we have*

(17) $$\begin{array}{cc} w_{k|k}^{i,(a^{i},0)} & =w_{k|k-1}^{i,a^{i}}\left(1-r_{k|k-1}^{i,a^{i}}+r_{k|k-1}^{i,a^{i}}\int \left(1-p_{D,k}\left(x\right)\right)p_{k|k-1}^{i,a^{i}}\left(x\right)dx\right), \end{array}$$

(18) $$\begin{array}{cc} r_{k|k}^{i,(a^{i},0)} & =\frac{r_{k|k-1}^{i,a^{i}}\int \left(1-p_{D,k}\left(x\right)\right)p_{k|k-1}^{i,a^{i}}\left(x\right)dx}{1-r_{k|k-1}^{i,a^{i}}+r_{k|k-1}^{i,a^{i}}\int \left(1-p_{D,k}\left(x\right)\right)p_{k|k-1}^{i,a^{i}}\left(x\right)dx}, \end{array}$$

(19) $$\begin{array}{cc} p_{k|k}^{i,(a^{i},0)}\left(x\right) & =\frac{\left(1-p_{D,k}\left(x\right)\right)p_{k|k-1}^{i,a^{i}}\left(x\right)}{\int \left(1-p_{D,k}\left(x\right)\right)p_{k|k-1}^{i,a^{i}}\left(x\right)dx}, \end{array}$$

*where $p_{D,k}\left(x\right)$ denotes the probability of detection. Under the measurement association hypothesis, the updated parameters are given by*

(20) $$\begin{array}{cc} w_{k|k}^{i,(a^{i},j)} & =\frac{w_{k|k-1}^{i,a^{i}}r_{k|k-1}^{i,a^{i}}\int p_{D,k}\left(x\right)l_{k}\left(z_{k}^{j}|x\right)p_{k|k-1}^{i,a^{i}}\left(x\right)dx}{\kappa_{k}\left(z_{k}^{j}\right)}, \end{array}$$

(21) $$\begin{array}{cc} r_{k|k}^{i,(a^{i},j)} & =1, \end{array}$$

(22) $$\begin{array}{cc} p_{k|k}^{i,(a^{i},j)}\left(x\right) & =\frac{p_{D,k}\left(x\right)l_{k}\left(z_{k}^{j}|x\right)p_{k|k-1}^{i,a^{i}}\left(x\right)}{\int p_{D,k}\left(x\right)l_{k}\left(z_{k}^{j}|x\right)p_{k|k-1}^{i,a^{i}}\left(x\right)dx}, \end{array}$$

*where $l_{k}\left(z|x\right)$ denotes the single-target likelihood function and $\kappa_{k}\left(z\right)$ denotes the intensity of a Poisson RFS modeling false alarms.*

It has been shown in [46] that the MBM recursion stated in the above propositions admits a closed-form solution under the following assumptions:

**Assumption** **1.** *The single-target state transition density $\varphi_{k+1|k}\left(x|x^{\prime }\right)$ satisfies*

(23) $$\begin{array}{c} \varphi_{k+1|k}\left(x|x^{\prime }\right)=N\left(x;F_{k}x^{\prime },Q_{k}\right), \end{array}$$

*where $F_{k}$ and $Q_{k}$ are the state transition matrix and process noise covariance matrix, respectively.*

**Assumption** **2.** *The single-target measurement likelihood function $l_{k}\left(z|x\right)$ satisfies*

(24) $$\begin{array}{c} l_{k}\left(z|x\right)=N\left(z;H_{k}x,R_{k}\right), \end{array}$$

*where $H_{k}$ is the measurement matrix and $R_{k}$ is the measurement noise covariance matrix.*

**Assumption** **3.** *The probabilities of survival and detection are constants, i.e.,*

(25) $$\begin{array}{c} p_{S,k}\left(x\right)=p_{S},\;\;p_{D,k}\left(x\right)=p_{D}. \end{array}$$

**Assumption** **4.** *Each BC in the predicted and filtering MBM densities has a Gaussian spatial density:*

(26) $$\begin{array}{c} p_{t|k}^{i,a^{i}}\left(x\right)=N\left(x;m_{t|k}^{i,a^{i}},P_{t|k}^{i,a^{i}}\right). \end{array}$$

The following propositions show how the parameters characterizing MBM densities are analytically propagated as time progresses.

**Proposition** **3** (prediction). *The prediction of parameters characterizing the filtering density is given in (10)–(16). For each surviving BC $i\in \left\{1,\cdots ,n_{k|k}\right\}$, we have*

(27) $$\begin{array}{cc} w_{k+1|k}^{i,a^{i}} & =w_{k|k}^{i,a^{i}}, \end{array}$$

(28) $$\begin{array}{cc} r_{k+1|k}^{i,a^{i}} & =p_{S}r_{k|k}^{i,a^{i}}, \end{array}$$

(29) $$\begin{array}{cc} p_{k+1|k}^{i,a^{i}}\left(x\right) & =N\left(x;m_{k+1|k}^{i,a^{i}},P_{k+1|k}^{i,a^{i}}\right), \end{array}$$

*where*

(30) $$\begin{array}{cc} m_{k+1|k}^{i,a^{i}} & =F_{k}m_{k|k}^{i,a^{i}}, \end{array}$$

(31) $$\begin{array}{cc} P_{k+1|k}^{i,a^{i}} & =F_{k}P_{k|k}^{i,a^{i}}F_{k}^{⊤}+Q_{k}, \end{array}$$

*For each newborn BC $i\in \left\{n_{k|k}+1,\cdots ,n_{k+1|k}\right\}$, we have*

(32) $$\begin{array}{cc} a^{i} & =(k+1,i-n_{k|k}), \end{array}$$

(33) $$\begin{array}{cc} w_{k+1|k}^{i,a^{i}} & =1, \end{array}$$

(34) $$\begin{array}{cc} r_{k+1|k}^{i,a^{i}} & =r_{k+1}^{b,i-n_{k|k}}, \end{array}$$

(35) $$\begin{array}{cc} p_{k+1|k}^{i,a^{i}}\left(x\right) & =p_{k+1}^{b,i-n_{k|k}}\left(x\right)=N\left(x;m_{k+1}^{b,i-n_{k|k}},P_{k+1}^{b,i-n_{k|k}}\right), \end{array}$$

*where the quantities $r_{k+1}^{b,i-n_{k|k}}$, $m_{k+1}^{b,i-n_{k|k}}$, and $P_{k+1}^{b,i-n_{k|k}}$ are given birth model parameters.*

**Proposition** **4** (update). *For each BC $i\in \left\{1,\cdots ,n_{k|k}\right\}$ with single-target hypothesis $a^{i}$, the updated parameters for the misdetection case are given in (18) and (19), which simplify as*

(36) $$\begin{array}{cc} \; & w_{k|k}^{i,(a^{i},0)}=w_{k|k-1}^{i,a^{i}}\left(1-r_{k|k-1}^{i,a^{i}}+\left(1-p_{D}\right)r_{k|k-1}^{i,a^{i}}\right), \end{array}$$

(37) $$\begin{array}{cc} \; & r_{k|k}^{i,(a^{i},0)}=\frac{\left(1-p_{D}\right)r_{k|k-1}^{i,a^{i}}}{1-r_{k|k-1}^{i,a^{i}}+\left(1-p_{D}\right)r_{k|k-1}^{i,a^{i}}}, \end{array}$$

(38) $$\begin{array}{cc} \; & p_{k|k}^{i,(a^{i},0)}\left(x\right)=p_{k|k-1}^{i,a^{i}}\left(x\right)=N\left(x;m_{k|k-1}^{i,a^{i}},P_{k|k-1}^{i,a^{i}}\right). \end{array}$$

*The updated parameters for the measurement association case are given in (20)–(22), which simplify as*

(39) $$\begin{array}{cc} w_{k|k}^{i,(a^{i},j)} & =\frac{w_{k|k-1}^{i,a^{i}}r_{k|k-1}^{i,a^{i}}p_{D}q_{k}^{i,(a^{i},j)}\left(z_{k}^{j}\right)}{\kappa_{k}\left(z_{k}^{j}\right)}, \end{array}$$

(40) $$\begin{array}{cc} r_{k|k}^{i,(a^{i},j)} & =1, \end{array}$$

(41) $$\begin{array}{cc} p_{k|k}^{i,(a^{i},j)}\left(x\right) & =N\left(x;m_{k|k}^{i,(a^{i},j)},P_{k|k}^{i,(a^{i},j)}\right), \end{array}$$

*where*

(42) $$\begin{array}{cc} q_{k}^{i,(a^{i},j)}\left(z_{k}^{j}|\xi \right) & =N\left(z_{k}^{j};H_{k}m_{k|k-1}^{i,a^{i}},S_{k|k-1}^{i,a^{i}}\right), \end{array}$$

(43) $$\begin{array}{cc} m_{k|k}^{i,(a^{i},j)} & =m_{k|k-1}^{i,a^{i}}+K_{k|k}^{i,a^{i}}\left(z_{k}^{j}-H_{k}m_{k|k-1}^{i,a^{i}}\right), \end{array}$$

(44) $$\begin{array}{cc} P_{k|k}^{i,(a^{i},j)} & =P_{k|k-1}^{i,a^{i}}-K_{k|k}^{i,a^{i}}S_{k|k-1}^{i,a^{i}}{\left(K_{k|k}^{i,a^{i}}\right)}^{⊤}, \end{array}$$

(45) $$\begin{array}{cc} S_{k|k}^{i,a^{i}} & =H_{k}P_{k|k-1}^{i,a^{i}}H_{k}^{⊤}+R_{k}, \end{array}$$

(46) $$\begin{array}{cc} K_{k|k}^{i,a^{i}} & =P_{k|k-1}^{i,a^{i}}H_{k}^{⊤}{\left(S_{k|k-1}^{i,a^{i}}\right)}^{-1}. \end{array}$$

After the prediction and update steps, the pruning of global hypotheses and BCs is further required to guarantee the computing efficiency. These procedures are detailed in [46] and, thus, will not be shown here for clarity. Moreover, the estimators given in [7] can be straightforwardly employed to extract multitarget state estimates.

## 4. Robust MMTT under Glint Noise

The Gaussian MBM filter stated in the previous section relies on both the single-model and Gaussian noise assumptions. In some practical applications, these assumptions are no longer valid. On the one hand, target maneuvers are inevitable, and it is sufficient to describe the target motion mode by a single model. On the other hand, if a target of interest is observed by a radar system, non-Gaussian glint noise may occur as a result of changes in the target aspect toward the radar [47], and exact noise statistics are generally not available. In this section, we will show how the Gaussian MBM filter is tailored to accommodate maneuvering targets in the context of glint measurement noise with unknown statistics. Specifically, in order to capture the real-time maneuvers of targets, a set of models is assumed for possible motion modes of each BC representing a single-target hypothesis. The switching among models follows a homogeneous Markov process with transition probability $t_{k+1|k}\left(\xi^{\prime }|\xi \right)$, where $\xi^{\prime },\xi \in M$ and M denotes the discrete set of model labels. In addition, the ML distribution is employed to model the measurement noise. As a result, the likelihood function $l_{k}\left(z|x\right)$ in (24) now becomes

(47) $$\begin{array}{c} l_{k}\left(z|x\right)=\mathrm{ML}\left(z;H_{k}x,R_{k}\right). \end{array}$$

For the sake of convenience, we make use of an equivalent form of the likelihood function $l_{k}\left(z|x\right)$ as

(48) $$l\left(z_{k}|x_{k}\right)=\mathrm{ML}\left(z_{k};H_{k}x_{k},R_{k}\right),$$

where ML(·;m,P) denotes the ML PDF of the form (6). According to (7) and (8), $l(z_{k}|x_{k})$ can be reformulated as

(49) $$\begin{array}{cc} p(z_{k}|\zeta_{k}) & =N(z_{k};H_{k}x_{k},\zeta_{k}R_{k}), \end{array}$$

(50) $$\begin{array}{cc} p(\zeta_{k}) & =E(\zeta_{k};\lambda_{0}). \end{array}$$

The prior distribution of $R_{k}$ is selected as

(51) $$\begin{array}{c} p\left(R_{k}\right)=\mathrm{IW}\left(R_{k};\Psi_{0},\nu_{0}\right). \end{array}$$

The rationality behind such a selection is that the IW distribution is the conjugate prior of a positive definite matrix [48].

The following propositions show how the unknown noise statistics are adaptively learned by means of the VB approximation accompanied by the IMM-MBM filtering.

**Proposition** **5** (prediction). *The predicted parameters for each surviving BC $i\in \left\{1,\cdots ,n_{k|k}\right\}$ and each model with label ξ∈M are given by*

(52) $$\begin{array}{cc} w_{k+1|k}^{i,a^{i}}\left(\xi \right) & =w_{k|k}^{i,a^{i}}\left(\xi \right), \end{array}$$

(53) $$\begin{array}{cc} r_{k+1|k}^{i,a^{i}}\left(\xi \right) & =p_{S}r_{k|k}^{i,a^{i}}\left(\xi \right), \end{array}$$

(54) $$\begin{array}{cc} p_{k+1|k}^{i,a^{i}}\left(x,\xi \right) & =N\left(x;m_{k+1|k}^{i,a^{i}}\left(\xi \right),P_{k+1|k}^{i,a^{i}}\left(\xi \right)\right), \end{array}$$

*where*

(55) $$\begin{array}{cc} m_{k+1|k}^{i,a^{i}}\left(\xi \right) & =F_{k}\left(\xi \right){\bar{m}}_{k|k}^{i,a^{i}}\left(\xi \right), \end{array}$$

(56) $$\begin{array}{cc} P_{k+1|k}^{i,a^{i}}\left(\xi \right) & =F_{k}\left(\xi \right){\bar{P}}_{k|k}^{i,a^{i}}\left(\xi \right)F_{k}{\left(\xi \right)}^{⊤}+Q_{k}\left(\xi \right), \end{array}$$

(57) $$\begin{array}{cc} {\bar{m}}_{k|k}^{i,a^{i}}\left(\xi \right) & =\sum_{\xi^{\prime }\in M}\mu_{k}^{i,a^{i}}\left(\xi^{\prime }|\xi \right)m_{k|k}^{i,a^{i}}\left(\xi^{\prime }\right), \end{array}$$

(58) $$\begin{array}{cc} {\bar{P}}_{k|k}^{i,a^{i}}\left(\xi \right) & =\sum_{\xi^{\prime }\in M}\mu_{k}^{i,a^{i}}\left(\xi^{\prime }|\xi \right)\left(P_{k|k}^{i,a^{i}}\left(\xi^{\prime }\right)+\Omega_{k|k}^{i,a^{i}}\left(\xi^{\prime }|\xi \right)\right), \end{array}$$

(59) $$\begin{array}{cc} \Omega_{k|k}^{i,a^{i}}\left(\xi^{\prime }|\xi \right) & =\left(m_{k|k}^{i,a^{i}}\left(\xi^{\prime }\right)-{\bar{m}}_{k|k}^{i,a^{i}}\left(\xi \right)\right){\left(m_{k|k}^{i,a^{i}}\left(\xi^{\prime }\right)-{\bar{m}}_{k|k}^{i,a^{i}}\left(\xi \right)\right)}^{⊤}, \end{array}$$

(60) $$\begin{array}{cc} \mu_{k}^{i,a^{i}}\left(\xi^{\prime }|\xi \right) & =\frac{t_{k+1|k}\left(\xi^{\prime }|\xi \right)\mu_{k}^{i,a^{i}}\left(\xi^{\prime }\right)}{\mu_{k+1|k}^{i,a^{i}}\left(\xi \right)}, \end{array}$$

(61) $$\begin{array}{cc} \mu_{k+1|k}^{i,a^{i}}\left(\xi \right) & =\sum_{\xi^{\prime }\in M}t_{k+1|k}\left(\xi^{\prime }|\xi \right)\mu_{k}^{i,a^{i}}\left(\xi^{\prime }\right). \end{array}$$

*Here, $\mu_{k}^{i,a^{i}}\left(\xi^{\prime }|\xi \right)$ is the mixing weight and $\mu_{k+1|k}^{i,a^{i}}\left(\xi \right)$ is the predicted model probability. For each newborn BC $i\in \left\{n_{k|k}+1,\cdots ,n_{k+1|k}\right\}$, the corresponding parameters are given by*

(62) $$\begin{array}{cc} a^{i} & =(k+1,i-n_{k|k}), \end{array}$$

(63) $$\begin{array}{cc} w_{k+1|k}^{i,a^{i}}\left(\xi \right) & =1, \end{array}$$

(64) $$\begin{array}{cc} r_{k+1|k}^{i,a^{i}}\left(\xi \right) & =r_{k+1}^{b,i-n_{k|k}}\left(\xi \right), \end{array}$$

(65) $$\begin{array}{cc} p_{k+1|k}^{i,a^{i}}\left(x,\xi \right) & =N\left(x;m_{k+1}^{b,i-n_{k|k}}\left(\xi \right),P_{k+1}^{b,i-n_{k|k}}\left(\xi \right)\right). \end{array}$$

*Note that the birth parameters $r_{k+1}^{b,i-n_{k|k}}\left(\xi \right)$, $m_{k+1}^{b,i-n_{k|k}}\left(\xi \right)$, and $P_{k+1}^{b,i-n_{k|k}}\left(\xi \right)$ are known a priori.*

**Proposition** **6** (update). *For the i-th BC and model with label ξ∈M, under the misdetection hypothesis, the updated parameters are given by*

(66) $$\begin{array}{cc} w_{k|k}^{i,(a^{i},0)}\left(\xi \right) & =w_{k|k-1}^{i,a^{i}}\left(\xi \right)\left(1-r_{k|k-1}^{i,a^{i}}\left(\xi \right)+\left(1-p_{D}\right)r_{k|k-1}^{i,a^{i}}\left(\xi \right)\right), \end{array}$$

(67) $$\begin{array}{cc} r_{k|k}^{i,(a^{i},0)}\left(\xi \right) & =\frac{\left(1-p_{D}\right)r_{k|k-1}^{i,a^{i}}\left(\xi \right)}{1-r_{k|k-1}^{i,a^{i}}\left(\xi \right)+\left(1-p_{D}\right)r_{k|k-1}^{i,a^{i}}\left(\xi \right)}, \end{array}$$

(68) $$\begin{array}{cc} p_{k|k}^{i,(a^{i},0)}\left(x,\xi \right) & =N\left(x;m_{k|k-1}^{i,a^{i}}\left(\xi \right),P_{k|k-1}^{i,a^{i}}\left(\xi \right)\right). \end{array}$$

*The relative parameters for the measurement association hypothesis are given by*

(69) $$\begin{array}{cc} w_{k|k}^{i,(a^{i},j)}\left(\xi \right) & =\frac{w_{k|k-1}^{i,a^{i}}\left(\xi \right)r_{k|k-1}^{i,a^{i}}\left(\xi \right)p_{D}q_{k}^{i,(a^{i},j)}\left(z_{k}^{j}|\xi \right)}{\kappa_{k}\left(z_{k}^{j}\right)}, \end{array}$$

(70) $$\begin{array}{cc} r_{k|k}^{i,(a^{i},j)}\left(\xi \right) & =1, \end{array}$$

(71) $$\begin{array}{cc} p_{k|k}^{i,(a^{i},j)}\left(x,\xi \right) & =N\left(x;m_{k|k}^{i,(a^{i},j)}\left(\xi \right),P_{k|k}^{i,(a^{i},j)}\left(\xi \right)\right), \end{array}$$

*where $m_{k|k}^{i,(a^{i},j)}\left(\xi \right)$, $P_{k|k}^{i,(a^{i},j)}\left(\xi \right)$, and $q_{k}^{i,(a^{i},j)}\left(z_{k}^{j}|\xi \right)$ are obtained by*

(72) $$\begin{array}{c} \left[m_{k|k}^{i,(a^{i},j)}\left(\xi \right),P_{k|k}^{i,(a^{i},j)}\left(\xi \right),q_{k}^{i,(a^{i},j)}\left(z_{k}^{j}|\xi \right)\right]=\mathrm{VB}\left(m_{k|k-1}^{i,a^{i}}\left(\xi \right),P_{k|k-1,r}^{i,a^{i}},H_{k},\Psi_{0},\nu_{0},\lambda_{0},N\right). \end{array}$$

*Here, [·]=VB(·) denotes the VB approximation and N denotes the number of VB iterations.*

The updated model probability for the misdetection hypothesis case denoted by $\mu_{k}^{i,(a^{i},0)}\left(\xi \right)$ equals $\mu_{k|k-1}^{i,a^{i}}\left(\xi \right)$, while the updated model probability for the measurement association hypothesis case denoted by $\mu_{k}^{i,(a^{i},j)}\left(\xi \right)$ is calculated by

(73) $$\begin{array}{c} \mu_{k}^{i,(a^{i},j)}\left(\xi \right)=\frac{\mu_{k|k-1}^{i,a^{i}}\left(\xi \right)q_{k}^{i,(a^{i},j)}\left(z_{k}^{j}|\xi \right)}{\sum_{\xi \in M}\mu_{k|k-1}^{i,a^{i}}\left(\xi \right)q_{k}^{i,(a^{i},j)}\left(z_{k}^{j}|\xi \right)}. \end{array}$$

Moreover, the model-dependent quantities are then combined to obtain the final estimates of the *i*-th BC according to

(74) $$\begin{array}{cc} w_{k|k}^{i,(a^{i},j)} & =\sum_{\xi \in M}w_{k|k}^{i,(a^{i},j)}\left(\xi \right)\mu_{k}^{i,(a^{i},j)}\left(\xi \right), \end{array}$$

(75) $$\begin{array}{cc} r_{k|k}^{i,(a^{i},j)} & =\sum_{\xi \in M}r_{k|k}^{i,(a^{i},j)}\left(\xi \right)\mu_{k}^{i,(a^{i},j)}\left(\xi \right), \end{array}$$

(76) $$\begin{array}{cc} m_{k|k}^{i,(a^{i},j)} & =\sum_{\xi \in M}m_{k|k}^{i,(a^{i},j)}\left(\xi \right)\mu_{k}^{i,(a^{i},j)}\left(\xi \right), \end{array}$$

(77) $$\begin{array}{cc} P_{k|k}^{i,(a^{i},j)} & =\sum_{\xi \in M}\mu_{k}^{i,(a^{i},j)}\left(\xi \right)\left(P_{k|k}^{i,(a^{i},j)}\left(\xi \right)+\Sigma_{k|k}^{i,(a^{i},j)}\left(\xi \right)\right), \end{array}$$

(78) $$\begin{array}{cc} \Sigma_{k|k}^{i,(a^{i},j)}\left(\xi \right) & =\left(m_{k|k}^{i,(a^{i},j)}-m_{k|k}^{i,(a^{i},j)}\left(\xi \right)\right){\left(m_{k|k}^{i,(a^{i},j)}-m_{k|k}^{i,(a^{i},j)}\left(\xi \right)\right)}^{⊤}. \end{array}$$

Note that the pruning and estimate-extraction procedures are needed after the prediction and update steps. They are the same as in the standard MBM filter. In addition, the above IMM-MBM filter is not restricted to the linear dynamic and measurement models. It can accommodate nonlinear models using approximate strategies such as the unscented transform [49] and spherical–radial cubature rule [50].

Now, we specify the VB approximation adopted in (72). Without loss of generality, we consider a notationally convenient form:

(79) $$\begin{array}{c} \left[m_{k|k},P_{k|k},q_{k|k}\left(z_{k}\right)\right]=\mathrm{VB}\left(m_{k|k-1},P_{k|k-1},H_{k},\Psi_{0},\nu_{0},\lambda_{0},N\right). \end{array}$$

For the hierarchical measurement model given in (49)–(51), an analytic solution of the joint posterior PDF $p(\Lambda_{k}|z_{1:k})$ is not available, where $\Lambda_{k}≜\left\{x_{k},R_{k},\zeta_{k}\right\}$ and $z_{1:k}$ denotes the measurement sequence accumulated to time *k*. Consequently, the VB method is exploited to obtain an approximate solution:

$$\begin{array}{c} p\left(\Lambda_{k}|z_{1:k}\right)\approx q\left(x_{k}\right)q\left(R_{k}\right)q\left(\zeta_{k}\right) \end{array}$$

such that $\mathrm{KLD}\left(q\left(x_{k}\right)q\left(R_{k}\right)q\left(\zeta_{k}\right)\left|\right|p\left(\Lambda_{k}|z_{1:k}\right)\right)$ is minimized, where $\mathrm{KLD}\left(\cdot ||\cdot \right)$ is the Kullback–Leibler divergence (KLD), defined as

(80) $$\begin{array}{c} \mathrm{KLD}\left(f||g\right)=\int f\left(x\right)\log\frac{f(x)}{g(x)}dx. \end{array}$$

Each term of the approximated posterior PDF satisfies

(81) $$\begin{array}{c} \log q\left(\vartheta \right)=E_{\Lambda_{k}^{-\vartheta }}\left[p\left(\Lambda_{k},z_{1:k}\right)\right]+c_{\vartheta }, \end{array}$$

where $\Lambda_{k}^{-\vartheta }\cup \left\{\vartheta \right\}≜\Lambda_{k}$ and $c_{\vartheta }$ is a ϑ-independent constant. To address the mutual coupling of the variational parameter, a fixed-point iteration scheme is adopted to obtain an approximation of q(ϑ) by iteratively solving (81). This means that q(ϑ) is updated as $q^{\left(d+1\right)}\left(\vartheta \right)$ by exploiting $q^{\left(d\right)}\left(\Lambda_{k}^{-\vartheta }\right)$ to compute the expectation in (81) at the (d+1)-th iteration. Using Bayes’ theorem, the joint PDF $p(\Lambda_{k},z_{1:k})$ can be formed as

(82) $$\begin{array}{cc} p(\Lambda_{k},z_{1:k})= & N\left(z_{k};H_{k}x_{k},\zeta_{k}R_{k})\mathrm{IW}(R_{k};\Psi_{0},\nu_{0}\right)\\ & \times N\left(x_{k};m_{k|k-1},P_{k|k-1}\right)E\left(\zeta_{k};\lambda_{0}\right)p\left(z_{1:k}\right). \end{array}$$

**Proposition** **7.** *Let $\vartheta =x_{k}$, and plugging (82) into (81), $q^{\left(d+1\right)}\left(x_{k}\right)$ is updated as a Gaussian PDF, i.e.,*

(83) $$\begin{array}{c} q^{\left(d+1\right)}\left(x_{k}\right)=N\left(x_{k};m_{k|k}^{\left(d+1\right)},P_{k|k}^{\left(d+1\right)}\right), \end{array}$$

*where*

(84) $$\begin{array}{cc} m_{k|k}^{\left(d+1\right)} & =m_{k|k-1}+G_{k|k}^{\left(d+1\right)}\left(z_{k}-H_{k}m_{k|k-1}\right), \end{array}$$

(85) $$\begin{array}{cc} P_{k|k}^{\left(d+1\right)} & =\left(I_{n_{x}}-G_{k|k}^{\left(d+1\right)}H_{k}\right)P_{k|k-1}. \end{array}$$

*Here, $n_{z}$ denotes the dimensions of measurement vector $z_{k}$ and $I_{n}$ denotes the n×n identity matrix, and the Kalman gain $G_{k|k}^{\left(d+1\right)}$ is calculated by*

(86) $$\begin{array}{c} G_{k|k}^{\left(d+1\right)}=P_{k|k-1}H_{k}^{⊤}{\left[H_{k}P_{k|k-1}H_{k}^{⊤}+{\tilde{R}}_{k}^{\left(d\right)}\right]}^{-1}, \end{array}$$

*where the modified measurement noise covariance matrix ${\tilde{R}}_{k}^{\left(d\right)}$ is of the form*

(87) $$\begin{array}{c} {\tilde{R}}_{k}^{\left(d\right)}={\left(E^{\left(d\right)}\left[\zeta_{k}^{-1}\right]\right)}^{-1}{\left(E^{\left(d\right)}\left[R_{k}^{-1}\right]\right)}^{-1}. \end{array}$$

**Proof.**  See Appendix A.    □

**Proposition** **8.** *Let $\vartheta =\zeta_{k}$, and plugging (82) into (81), $q^{\left(d+1\right)}\left(\zeta_{k}\right)$ is updated as a GIG PDF, i.e.,*

(88) $$\begin{array}{c} q^{\left(d+1\right)}\left(\zeta_{k}\right)=\mathrm{GIG}\left(\zeta_{k};a_{k|k}^{\left(d+1\right)},b_{k|k}^{\left(d+1\right)},c_{k|k}^{\left(d+1\right)}\right), \end{array}$$

*where*

(89) $$\begin{array}{cc} a_{k|k}^{\left(d+1\right)} & =\frac{1}{2}\mathrm{tr}\left(U_{k|k}^{\left(d+1\right)}E^{\left(d\right)}\left[R_{k}^{-1}\right]\right), \end{array}$$

(90) $$\begin{array}{cc} b_{k|k}^{\left(d+1\right)} & =\lambda_{0}, \end{array}$$

(91) $$\begin{array}{cc} c_{k|k}^{\left(d+1\right)} & =1-\frac{n_{z}}{2}. \end{array}$$

*The auxiliary parameter $U_{k|k}^{\left(d+1\right)}$ is given by*

(92) $$\begin{array}{c} U_{k|k}^{\left(d+1\right)}=E^{\left(d+1\right)}\left[\left(z_{k}-H_{k}x_{k}\right){\left(z_{k}-H_{k}x_{k}\right)}^{⊤}\right]. \end{array}$$

**Proof.**  See Appendix B.    □

**Proposition** **9.** *Let $\vartheta =R_{k}$, and plugging (82) into (81), $q^{\left(d+1\right)}\left(R_{k}\right)$ is updated as an IW PDF, i.e.,*

(93) $$\begin{array}{c} q^{\left(d+1\right)}\left(R_{k}\right)=\mathrm{IW}\left(R_{k};\Psi_{k|k}^{\left(d+1\right)},\nu_{k|k}^{\left(d+1\right)}\right), \end{array}$$

*where*

(94) $$\begin{array}{cc} \Psi_{k|k}^{\left(d+1\right)} & =\Psi_{0}+E^{\left(d+1\right)}\left[\zeta_{k}^{-1}\right]U_{k|k}^{\left(d+1\right)}, \end{array}$$

(95) $$\begin{array}{cc} \nu_{k|k}^{\left(d+1\right)} & =\nu_{0}+1. \end{array}$$

**Proof.**  See Appendix C.    □

To implement the fixed-point iteration, the required expectations need to be calculated as follows. Using (83), the auxiliary parameter $U_{k|k}^{\left(d+1\right)}$ in (92) is calculated by

(96) $$\begin{array}{c} U_{k|k}^{\left(d+1\right)}=\left(z_{k}-H_{k}m_{k|k}^{\left(d+1\right)}\right){\left(z_{k}-H_{k}m_{k|k}^{\left(d+1\right)}\right)}^{⊤}+H_{k}P_{k|k}^{\left(d+1\right)}H_{k}^{⊤}. \end{array}$$

Employing (88), the expectation $E^{\left(d+1\right)}\left[\zeta_{k}^{-1}\right]$ is computed as

(97) $$\begin{array}{c} E^{\left(d+1\right)}\left[\zeta_{k}^{-1}\right]=\frac{\sqrt{a_{k|k}^{\left(d+1\right)}}B_{c_{k|k}^{\left(d+1\right)}+1}\left(\sqrt{a_{k|k}^{\left(d+1\right)}b_{k|k}^{\left(d+1\right)}}\right)}{\sqrt{b_{k|k}^{\left(d+1\right)}}B_{c_{k|k}^{\left(d+1\right)}}\left(\sqrt{a_{k|k}^{\left(d+1\right)}b_{k|k}^{\left(d+1\right)}}\right)}. \end{array}$$

Utilizing (93), the expectation $E^{\left(d+1\right)}\left[R_{k}^{-1}\right]$ is computed as

(98) $$\begin{array}{c} E^{\left(d+1\right)}\left[R_{k}^{-1}\right]=\nu_{k|k}^{\left(d+1\right)}/\Psi_{k|k}^{\left(d+1\right)}. \end{array}$$

In addition, abiding by [29,31], the predictive likelihood $q_{k|k}\left(z_{k}^{i}\right)$ satisfies

(99) $$\begin{array}{c} \log q_{k|k}\left(z_{k}\right)=\mathrm{KLD}\left\{q\left(x_{k}\right)q\left(R_{k}\right)q\left(\zeta_{k}\right)\left|\right|p\left(\Lambda_{k}|z_{1:k}\right)\right\}+L\left(q\left(x_{k}\right)q\left(R_{k}\right)q\left(\zeta_{k}\right)\right). \end{array}$$

Since the first term on the right-hand side of (99) is minimized by the VB method, $L(q\left(x_{k}\right)$$q\left(R_{k}\right)q\left(\zeta_{k}\right))$ can be treated as the variational lower bound of $\log q_{k|k}\left(z_{k}\right)$. As a consequence, $q_{k|k}\left(z_{k}\right)$ can be approximately computed as

(100) $$\begin{array}{c} q_{k|k}\left(z_{k}\right)\approx \exp\left(L(q\left(x_{k}\right)q\left(R_{k}\right)q\left(\zeta_{k}\right))\right), \end{array}$$

where

(101) $$\begin{array}{cc} L\left(q\left(x_{k}\right)q\left(R_{k}\right)\right)q\left(\zeta_{k}\right) & =E\left[\log N\left(z_{k};H_{k}x_{k},\zeta_{k}R_{k}\right)\right)]+E\left[\log\mathrm{IW}\left(R_{k};\Psi_{0},\nu_{0}\right)\right]\\ & \;+E\left[\log N\left(x_{k};m_{k|k-1},P_{k|k-1}\right)\right]-E\left[\log\mathrm{IW}\left(R_{k};\Psi_{k|k},\nu_{k|k}\right)\right]\\ & \;-E\left[\log N\left(x_{k};m_{k|k},P_{k|k}\right)\right]+E\left[\log E\left(\zeta_{k};\lambda_{0}\right)\right]\\ & \;-E[\log\mathrm{GIG}\left(\zeta_{k};a_{k|k},b_{k|k},c_{k|k}\right)]. \end{array}$$

The explicit expression for $L(q\left(x_{k}\right)q\left(R_{k}\right)q\left(\zeta_{k}\right))$ can be found in Appendix D. A summary of the VB function of the form (79) is given in Algorithm 1, where ${\bar{R}}_{k}$ is the nominal measurement covariance matrix.
**Algorithm** **1:** A summary of the VB function.**Input:** $m_{k|k-1}$, $P_{k|k-1}$, $z_{k}$, $H_{k}$, *N*, $\Psi_{0}$, $\nu_{0}$, $\lambda_{0}$.1:Initialization: $E^{\left(0\right)}\left[\zeta_{k}^{-1}\right]=1$, $E^{\left(0\right)}\left[R_{k}^{-1}\right]={\bar{R}}_{k}^{-1}$.2:**for** d=0,1,2,⋯,N−1 **do**3:   Compute ${\tilde{R}}_{k}^{\left(d\right)}$ and $G_{k|k}^{\left(d+1\right)}$ using (87) and (86), respectively.4:   Update $q^{\left(d+1\right)}\left(x_{k}\right)$ as a Gaussian PDF using (83) with the mean vector $m_{k|k}^{\left(d+1\right)}$ and covariance matrix $P_{k|k}^{\left(d+1\right)}$ calculated by (84)–(85).5:   Compute $U_{k|k}^{\left(d+1\right)}$ using (96).6:   Update $q^{\left(d+1\right)}\left(\zeta_{k}\right)$ as a GIG PDF using (88) with shape parameters $a_{k|k}^{\left(d+1\right)}$, $b_{k|k}^{\left(d+1\right)}$, and $c_{k|k}^{\left(d+1\right)}$ calculated by (89)–(91).7:   Compute $E^{\left(d+1\right)}\left[\zeta_{k}^{-1}\right]$ using (97).8:   Update $q^{\left(d+1\right)}\left(R_{k}\right)$ as an IW PDF using (93) with the scale matrix $\Psi_{k|k}^{\left(d+1\right)}$ and DOF parameter $\nu_{k|k}^{\left(d+1\right)}$ calculated by (94) and (95), respectively.9:   Compute $E^{\left(d+1\right)}\left[R_{k}^{-1}\right]$ using (98).10:**end for**11:Set $m_{k|k}=m_{k|k}^{\left(N\right)}$, $P_{k|k}=P_{k|k}^{\left(N\right)}$, $a_{k|k}=a_{k|k}^{\left(N\right)}$, $b_{k|k}=b_{k|k}^{\left(N\right)}$, $c_{k|k}=c_{k|k}^{\left(N\right)}$, $\Psi_{k|k}=\Psi_{k|k}^{\left(N\right)}$, and $\nu_{k|k}=\nu_{k|k}^{\left(N\right)}$.12:Compute $q_{k|k}\left(z_{k}\right)$ using (100).**Output:** $m_{k|k}$, $P_{k|k}$, and $q_{k|k}\left(z_{k}\right)$.

## 5. Numerical Studies

A 2D tracking scenario involving four maneuvering targets is considered to demonstrate the performance of the proposed MMTT algorithm (hereafter refereed to as the ML-IMM-MBM filter). The target kinematic state $x_{k}$ is composed of position ${\left[p_{x,k},p_{y,k}\right]}^{⊤}$ and velocity ${\left[{\dot{p}}_{x,k},{\dot{p}}_{y,k}\right]}^{⊤}$, i.e., $x_{k}={\left[p_{x,k},{\dot{p}}_{x,k},p_{y,k},{\dot{p}}_{y,k}\right]}^{⊤}$. All targets follow the state dynamics:

$$\begin{array}{cc} x_{k}\; & =\left[\begin{array}{cccc} 1 & \frac{\sin(\omega T)}{\omega } & 0 & -\frac{1-\cos(\omega T)}{T}\\ 0 & \sin(\omega T) & 0 & -\sin(\omega T)\\ 0 & \frac{1-\cos(\omega T)}{T} & 1 & \frac{\sin(\omega T)}{T}\\ 0 & \sin(\omega T) & 0 & \cos(\omega T) \end{array}\right]x_{k-1}+w_{k} \end{array}$$

with different turn rates $\omega \in \left\{-10{}^{\circ}/s,0,10{}^{\circ}/s\right\}$, where T=1 s is the scan period and $w_{k}$ is the zero-mean Gaussian process noise with covariance matrix

$$\begin{array}{cc} Q_{k} & =\sigma_{w}^{2}\left[\begin{array}{cccc} \frac{T^{4}}{4} & \frac{T^{3}}{2} & 0 & 0\\ \frac{T^{3}}{2} & T^{2} & 0 & 0\\ 0 & 0 & \frac{T^{4}}{4} & \frac{T^{3}}{2}\\ 0 & 0 & \frac{T^{3}}{2} & T^{2} \end{array}\right]. \end{array}$$

More specifically, ω=0 corresponds to a constant velocity model indexed by M1 with $\sigma_{w}=10\;m/s^{2}$, while ω=−10°/s and ω=10°/s correspond to two different coordinate turn models indexed by M2 and M3, respectively. For models M2 and M3, $\sigma_{w}=20\text{°}m/s^{2}$. The model transition probability matrix is given by

$$\begin{array}{c} \left[t_{k+1|k}\left(\xi^{\prime }|\xi \right)\right]=\left[\begin{array}{ccc} 0.8 & 0.1 & 0.1\\ 0.1 & 0.8 & 0.1\\ 0.1 & 0.1 & 0.8 \end{array}\right]. \end{array}$$

**Figure 1:**
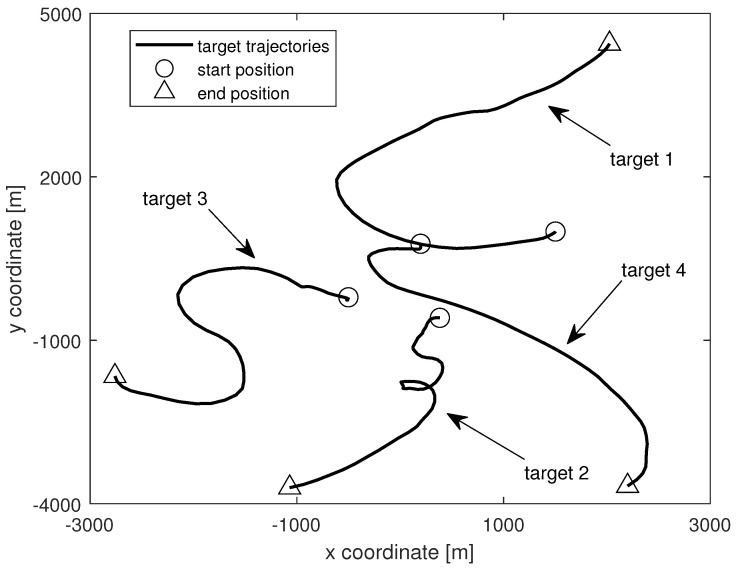
Truetrajectories of the targets.

The simulation lasted 80 s, corresponding to 80 scans. The dynamics of each target is given as follows. Target 1 appears at time k=1s and disappears at time k=60s. It follows model M2 during [1,25]s, model M1 during [26,40]s, and model M3 during [41,60]s. Target 2 appears at time k=10s and disappears at time k=70s. It follows model M3 during [10,30]s, model M2 during [31,60]s, and model M1 during [61,70]s. Target 3 appears at time k=20s and disappears at time k=80s. It follows model M1 during [20,40]s, model M3 during [41,60]s, and model M2 during [61,80]s. Target 4 appears at time k=30s and disappears at time k=80s. It follows model M3 during [30,50]s, model M1 during [51,70]s, and model M2 during [71,80]s. The true trajectories of the targets are plotted in Figure 1. The survival probability $p_{S}$ is 0.98 and the MB parameter set for newborn targets is represented by

$$\begin{array}{c} {\left\{{\left\{\left(r_{k+1}^{b,q}\left(\xi \right),p_{k+1}^{b,q}\left(x,\xi \right)\right)\right\}}_{\xi \in M}\right\}}_{q=1}^{4}, \end{array}$$

where $r_{k+1}^{b,q}\left(\xi \right)=0.1$ and $p_{k+1}^{b,q}\left(x,\xi \right)$ takes the form

$$\begin{array}{c} p_{k+1}^{b,q}\left(x,\xi \right)=N\left(x;m_{k+1}^{b,q}\left(\xi \right),P_{k+1}^{b}\left(\xi \right)\right), \end{array}$$

where

$$\begin{array}{cc} m_{k+1}^{b,1}\left(\xi \right) & ={\left[1500\;m;0;1000\;m;0\right]}^{⊤},\\ m_{k+1}^{b,2}\left(\xi \right) & ={\left[400\;m;0;-600\;m;0\right]}^{⊤},\\ m_{k+1}^{b,1}\left(\xi \right) & ={\left[-500\;m;0;-200\;m;0\right]}^{⊤},\\ m_{k+1}^{b,1}\left(\xi \right) & ={\left[200\;m;0;800\;m;0\right]}^{⊤},\\ P_{k+1}^{b}\left(\xi \right) & =\mathrm{diag}{\left({\left[10\;m,10\;m/s^{2},10\;m,10\;m/s^{2}\right]}^{⊤}\right)}^{2}. \end{array}$$

A radar observes the targets of interest and provides both the range and angle according to the measurement equation:

$$\begin{array}{c} z_{k}=\left[\begin{array}{c} \sqrt{{\left(p_{x,k}-p_{s,x}\right)}^{2}+{\left(p_{y,k}-p_{s,y}\right)}^{2}}\\ \arctan\left(\frac{p_{x,k}-p_{s,x}}{p_{y,k}-p_{s,y}}\right) \end{array}\right]+v_{k}, \end{array}$$

where ${\left[p_{s,x},p_{s,y}\right]}^{⊤}={\left[1000\;m,2000\;m\right]}^{⊤}$ denotes the location of the radar and $v_{k}$ is the glint measurement noise satisfying [31]

$$\begin{array}{c} v_{k}∼\gamma N\left(\cdot ;0_{2},\tau {\bar{R}}_{k}\right)+\left(1-\gamma \right)N\left(\cdot ;0_{2},{\bar{R}}_{k}\right). \end{array}$$

Here, $0_{n}$ denotes the *n*-dimensional zero vector, ${\bar{R}}_{k}=\mathrm{diag}{\left({\left[10\;m,5{}^{\circ}/s\right]}^{⊤}\right)}^{2}$ is the nominal measurement covariance matrix, τ=100 is a scale factor, and γ=0.1 is the glint probability. The probability of detection is $p_{D}=0.9$. Apart from target-originated measurements, at each scan, an average of 10 false alarms are also received by the sensor.

**Figure 2:**
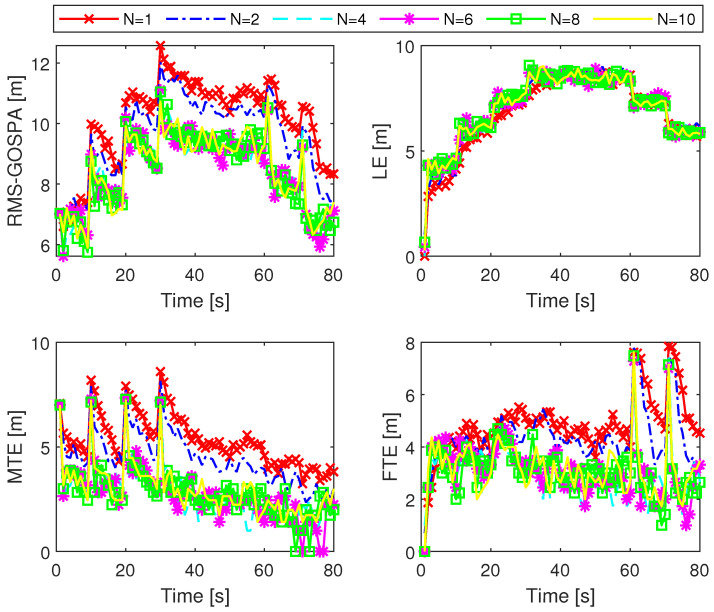
GOSPAE, LE, MTE, and FTE of the ML-IMM-MBM filter for different *N* with β=8.

**Figure 3:**
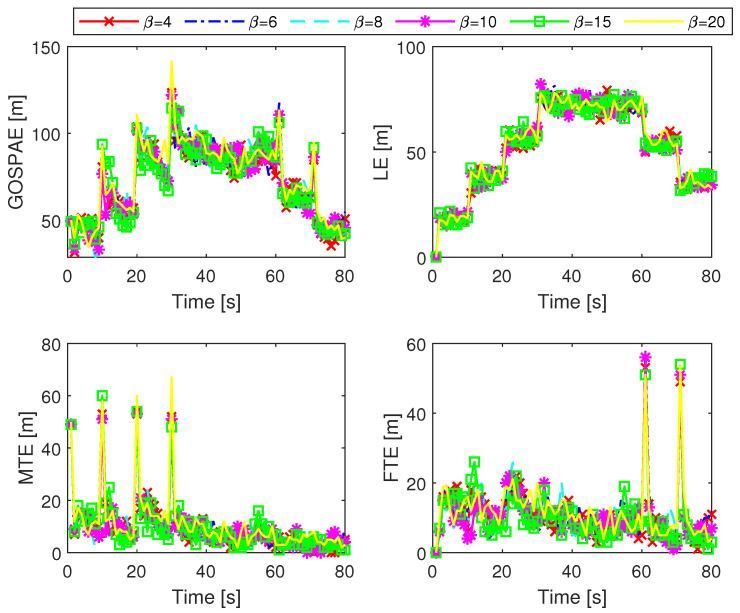
GOSPAE, LE, MTE, and FTE of the ML-IMM-MBM filter for different β with N=6.

We first tested the ML-IMM-MBM filter with 100 Monte Carlo (MC) trials. In each trial, the true target tracks remained unchanged, whereas the sensor measurements were randomly generated. In the ML-IMM-MBM filter, the prior parameters were configured as $\lambda_{0}=1$, $\nu_{0}=\beta$, and $\Psi_{k}=\beta {\bar{R}}_{k}$, where β is a user-defined parameter satisfying $\beta >n_{z}+1$. The thresholds for pruning, merging, and state extraction were set as $10^{-5}$, 4, and 0.4, respectively. In addition, the ML-IMM-MBM filter uses a maximum number of global hypotheses $N_{h}=100$ and adopts the unscented transform [49] to tackle measurement model nonlinearity. We evaluated the filter performance using the generalized optimal subpattern assignment (GOSPA) metric [51] with α=2, p=2, and c=10 m. The GOSPA error (GOSPAE) along with its decomposed localization error (LE), missed target error (MTE), and false target error (FTE) for different settings of the VB iteration number *N* are presented in Figure 2. It can be seen that the ML-IMM-MBM filter has a similar performance when N≥4. This can be attributed to the fast convergence of the VB approach [32]. The relevant results for different settings of the prior parameter β are shown in Figure 3. It can be seen that the ML-IMM-MBM filter behaves almost identically for varying parameter β, indicating excellent robustness to the prior parameter β.

**Figure 4:**
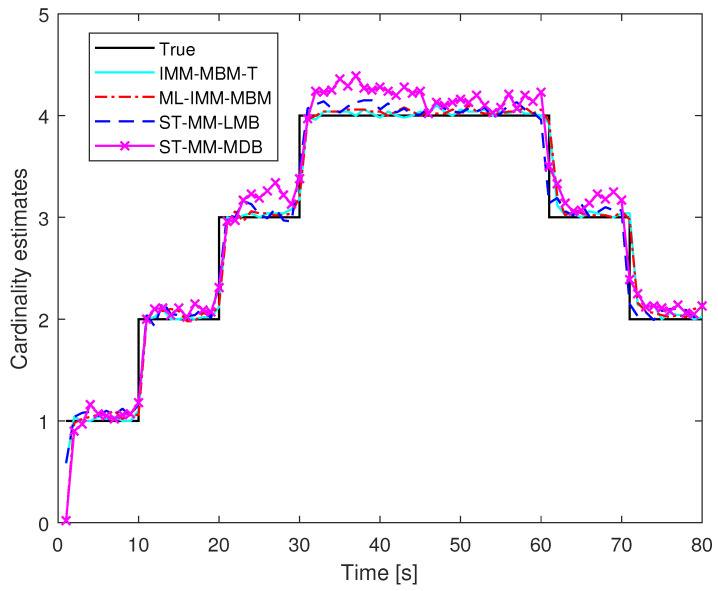
Cardinality estimates for filters under study.

**Figure 5:**
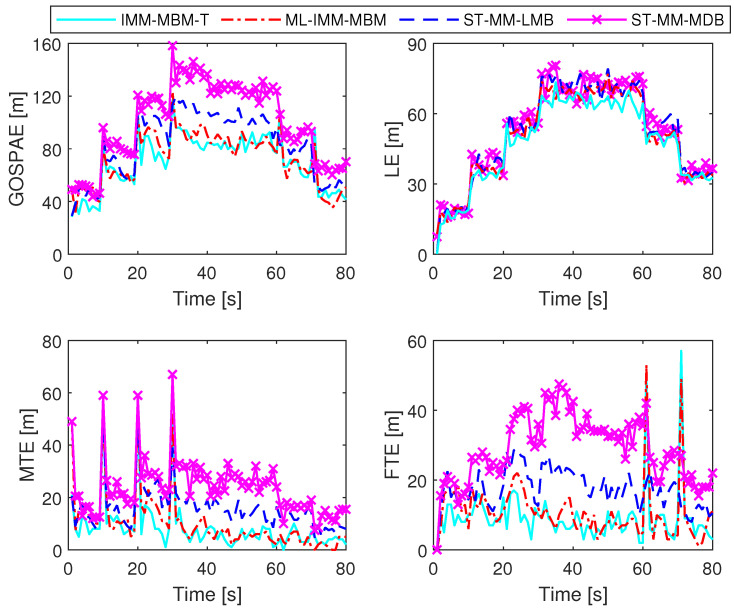
GOSPAE, LE, MTE, and FTE for filters under study.

**Figure 6:**
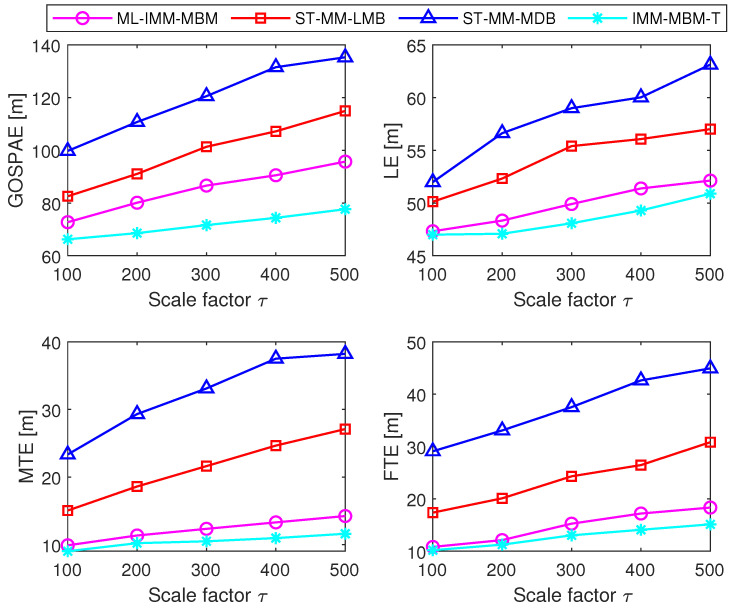
GOSPAE, LE, MTE, and FTE of different filters for varying scale factor.

**Figure 7:**
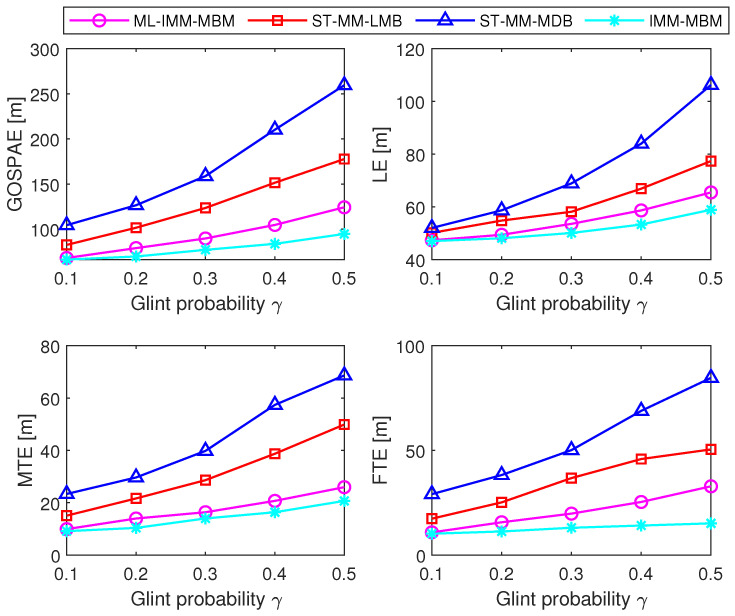
GOSPAE, LE, MTE, and FTE of different filters for varying glint probability.

We then compared the ML-IMM-MBM filter with the ST-MM-MDB filter, ST-MM-LMB filter, and the IMM-MBM filter with the true measurement noise covariance matrix (hereafter referred to as the IMM-MBM-T filter) with 100 MC trials. In the ML-IMM-MBM filter, the prior parameter β was set as β=8. Necessary parameters for the ST-MM-MDB filter and ST-MM-LMB filter were configured consistent with [33,34], respectively. In all filters under study, the VB iteration number *N* was set as N=6. Figure 4 and Figure 5 show the performance of these filters in terms of cardinality estimates and the GOSPA metric, respectively. It is shown that the ML-IMM-MBM filter is comparable to the MM-MBM-T filter, but outperforms the ST-MM-MDB and ST-MM-LMB filters. This can be attributed to two reasons. First, the MBM filter is an exact solution to the Bayes multitarget recursion (centerpiece of RFS-based MTT), while the MDB filter [52] and LMB filter [9] are approximate alternatives. Second, the ML-IMM-MBM filter models glint noise by the ML distribution, which gets rid of the selection of the DOF parameter, which is necessary in the ST-MM-MDB filter. The GOSPAE and its decomposition of different filters for varying scale factors and glint probability are shown in Figure 6 and Figure 7, respectively. It can be observed that the glint probability has a more significant influence on performance, and all the results indicate the superiority of the proposed ML-MM-MBM filters over the existing ST-MM-MDB and ST-MM-LMB filters.

## 6. Conclusions

In this article, we proposed a robust algorithm to address the problem of tracking multiple maneuvering targets with random appearances and disappearances from radar measurements corrupted by glint noise. Specifically, in order to capture the real-time maneuvers of targets, a set of models was assumed for the possible motion modes of each single-target hypothesis. Furthermore, the ML distribution was employed to model measurement noise, and the relevant likelihood function was formulated by a Gaussian scale form, leading to a hierarchical Gaussian model. Using this model, a robust IMM-MBM filter was derived based on the VB method, which can adaptively learn unknown noise statistics while filtering. In particular, the involved predicted likelihood was approximately calculated by means of the variational lower bound. The simulation results showed that our proposed algorithm not only possesses strong robustness to its freedom parameters, but also outperforms the existing ST-MM-MDB and ST-MM-LMB filters. For future work, we will extend the proposed algorithm to accommodate amplitude information under different SNRs and a network of cooperative sensors with limited sensing ranges.

## Nomenclature

x	Single-target state vector
z	Single-target measurement vector
X	Multitarget state RFS
Z	Multitarget measurement RFS
*k*	Discrete time step
$\varphi_{k+1|k}\left(\cdot |\cdot \right)$	Single-target state transition density
$a^{i}$	Single-target hypothesis for the *i*-th BC
a	Global hypothesis
Γ(·)	Gamma function
$n_{x}$	State vector dimension
$n_{z}$	Measurement vector dimension
$l_{k}\left(\cdot |\cdot \right)$	Measurement likelihood function
$p_{S,k}\left(\cdot \right)$	Probability of survival
$p_{D,k}\left(\cdot \right)$	Probability of detection
$\kappa_{k}\left(\cdot \right)$	Clutter intensity

## Appendix A

Employing (82), we have

(A1) $$\begin{array}{cc} \log p(\Lambda_{k},z_{1:k}) & =-\frac{1}{2\zeta_{k}}{\left(z_{k}-H_{k}x_{k}\right)}^{⊤}R_{k}^{-1}\left(z_{k}-H_{k}x_{k}\right)\\ & \;-\frac{1}{2}{\left(x_{k}-m_{k|k-1}\right)}^{⊤}P_{k|k-1}^{-1}\left(x_{k}-m_{k|k-1}\right)\\ & \;-\frac{1}{2}\mathrm{tr}\left(\Psi_{0}R_{k}^{-1}\right)-\frac{1}{2}\left(n_{z}+\nu_{0}+2\right)\log\left(\det\left(R_{k}\right)\right)\\ & \;-\lambda_{0}\zeta_{k}-\frac{n_{z}}{2}\log\zeta_{k}+c_{\Lambda_{k}}. \end{array}$$

Plugging $\vartheta =x_{k}$ into (81) and employing (A1) yield

(A2) $$\begin{array}{cc} \log q^{\left(d+1\right)}\left(x_{k}\right) & \propto -\frac{1}{2}{\left(x_{k}-m_{k|k-1}\right)}^{⊤}P_{k|k-1}^{-1}\left(x_{k}-m_{k|k-1}\right)\\ & \;-\frac{1}{2}{\left(z_{k}-H_{k}x_{k}\right)}^{⊤}E^{\left(d\right)}\left[\zeta_{k}^{-1}\right]E^{\left(d\right)}\left[R_{k}^{-1}\right]\left(z_{k}-H_{k}x_{k}\right). \end{array}$$

Exploiting (87) in (A2), $\log q^{\left(d+1\right)}\left(x_{k}\right)$ can be formulated as

(A3) $$\begin{array}{cc} \log q^{\left(d+1\right)}\left(x_{k}\right) & \propto \log N\left(x_{k};m_{k|k},P_{k|k}\right)+\log N\left(z_{k};H_{k}x_{k},{\tilde{R}}_{k}^{\left(d\right)}\right), \end{array}$$

Using (A3), $q^{\left(d+1\right)}\left(x_{k}\right)$ can be written as

(A4) $$\begin{array}{c} q^{\left(d+1\right)}\left(x_{k}\right)\propto N\left(x_{k};m_{k|k},P_{k|k}\right)N\left(z_{k};H_{k}x_{k},{\tilde{R}}_{k}^{\left(d\right)}\right). \end{array}$$

According to (A4), $q^{\left(d+1\right)}\left(x_{k}\right)$ can be updated as a Gaussian PDF as in (83), where the relevant parameters $m_{k|k}^{\left(d+1\right)}$ and $P_{k|k}^{\left(d+1\right)}$ can be computed using the measurement update of the standard Kalman filter as in (84) and (85).

## Appendix B

Plugging $\vartheta =\zeta_{k}$ into (81) and employing (A1) yield

(A5) $$\begin{array}{cc} \log q^{\left(d+1\right)}\left(\zeta_{k}\right) & \propto -\frac{1}{2\zeta_{k}}\mathrm{tr}\left(U_{k|k}^{\left(d+1\right)}E^{\left(d\right)}\left[R_{k}^{-1}\right]\right)-\frac{n_{z}}{2}\log\zeta_{k}-\lambda_{0}\zeta_{k}. \end{array}$$

Exploiting (89)–(91) in (A5), we have

(A6) $$\begin{array}{c} \log q^{\left(d+1\right)}\left(\zeta_{k}\right)\propto -\frac{a_{k|k}^{\left(d+1\right)}}{\zeta_{k}}-b_{k|k}^{\left(d+1\right)}\zeta_{k}-\left(c_{k|k}^{\left(d+1\right)}-1\right)\log\zeta_{k}. \end{array}$$

According to (A6), $q^{\left(d+1\right)}\left(\zeta_{k}\right)$ can be updated as a GIG PDF as in (88) with the relevant parameters $a_{k|k}^{\left(d+1\right)}$, $b_{k|k}^{\left(d+1\right)}$, and $c_{k|k}^{\left(d+1\right)}$ calculated by (89)–(91).

## Appendix C

Plugging $\vartheta =R_{k}$ into (81) and employing (A1) yield

(A7) $$\begin{array}{cc} \log q^{\left(d+1\right)}\left(R_{k}\right) & \propto -\frac{1}{2}\left(n_{z}+\nu_{0}+2\right)\log\left(\det(R_{k})\right)\\ & \;+\frac{1}{2}\mathrm{tr}\left(\left(E^{\left(d+1\right)}\left[\zeta_{k}^{-1}\right]U_{k|k}^{\left(d+1\right)}+\Psi_{0}\right)R_{k}^{-1}\right). \end{array}$$

Exploiting (94) and (95) in (A7), we have

(A8) $$\begin{array}{cc} \log q^{\left(d+1\right)}\left(R_{k}\right) & \propto -\frac{1}{2}\left(n_{z}+\nu_{k|k}^{\left(d+1\right)}+1\right)\log\left(\det\left(R_{k}\right)\right)+\frac{1}{2}\mathrm{tr}\left(\Psi_{k|k}^{\left(d+1\right)}R_{k}^{-1}\right). \end{array}$$

According to (A8), $q^{\left(d+1\right)}\left(R_{k}\right)$ can be updated as an IW PDF as in (93) with the relevant parameters $\Psi_{k|k}^{\left(d+1\right)}$ and $\nu_{k|k}^{\left(d+1\right)}$ calculated by (94) and (95).

## Appendix D

Each quality on the right-hand side of (101) is computed as follows:

(A9) $$\begin{array}{cc} E[\log N\left(z_{k};H_{k}x_{k},\zeta_{k}R_{k}\right))] & =-\frac{1}{2}\mathrm{tr}\left(E\left[\zeta_{k}^{-1}\right]E\left[R_{k}^{-1}\right]E\left[\left(z_{k}-H_{k}x_{k}\right){\left(z_{k}-H_{k}x_{k}\right)}^{⊤}\right]\right)\\ & \;-\frac{n_{z}}{2}\log\left(2\pi \right)-\frac{1}{2}E\left[\log\left(\det\left(R_{k}\right)\right)\right], \end{array}$$

where the expectation $E[\log\left(\det\left(R_{k}\right)\right)]$ is given by

(A10) $$\begin{array}{c} E\left[\log\left(\det\left(R_{k}\right)\right)\right]=\sum_{i=1}^{n_{z}}\psi \left(\frac{\nu_{k|k}+1-i}{2}\right)-n_{z}\log2+\log\det\left(\Psi_{k|k}\right), \end{array}$$

and

(A11) $$\begin{array}{cc} E[\left(z_{k}-H_{k}x_{k}\right){\left(z_{k}-H_{k}x_{k}\right)}^{⊤}]= & H_{k}P_{k|k}H_{k}^{⊤}+\left(z_{k}-H_{k}m_{k|k}\right){\left(z_{k}-H_{k}m_{k|k}\right)}^{⊤}. \end{array}$$

Here, ψ(·) denotes the digamma function.

Recall that, here, $\zeta_{k}$ and $R_{k}$ are exponential and IW distributed as in (50) and (51), respectively. Hence, the expectations $E[\zeta_{k}^{-1}]$ and $E[R_{k}^{-1}]$ are of the form

(A12) $$\begin{array}{c} E\left[\zeta_{k}^{-1}\right]=\lambda_{0},\;E\left[R_{k}^{-1}\right]=\left(\nu_{0}-n_{z}-1\right)\Psi_{0}^{-1}. \end{array}$$

(A13) $$\begin{array}{cc} E[\log N\left(x_{k};m_{k|k-1},P_{k|k-1}\right)] & =-\frac{1}{2}\log\left(\det\left(P_{k|k-1}\right)\right)-\frac{n_{x}}{2}\log\left(2\pi \right)\\ & \;-\mathrm{tr}\left(P_{k|k-1}^{-1}E\left[\left(x_{k}-m_{k|k-1}\right){\left(x_{k}-m_{k|k-1}\right)}^{⊤}\right]\right), \end{array}$$

where

(A14) $$\begin{array}{c} E\left[\left(x_{k}-m_{k|k-1}\right){\left(x_{k}-m_{k|k-1}\right)}^{⊤}\right]=P_{k|k}+\left(m_{k|k}-m_{k|k-1}\right){\left(m_{k|k}-m_{k|k-1}\right)}^{⊤}. \end{array}$$

(A15) $$\begin{array}{cc} E[\log\mathrm{IW}\left(R_{k};\Psi_{k|k},\nu_{k|k}\right)] & =-\frac{\nu_{k|k}}{2}\log\left(\det\left(\Psi_{k|k}\right)\right)-\frac{\nu_{k|k}+n_{z}+1}{2}E\left[\log\left(\det\left(R_{k}\right)\right)\right]\\ & \;-\frac{1}{2}\mathrm{tr}\left(\Psi_{k|k}E\left[R_{k}^{-1}\right]\right)-\frac{n_{z}\nu_{k|k}}{2}\log2\;-\log\Gamma_{n_{z}}\left(\frac{\nu_{k|k}}{2}\right), \end{array}$$

where the equalities $E[\log\left(\det\left(R_{k}\right)\right)]$ and $E[R_{k}^{-1}]$ are given in (A10) and (A12), respectively.

(A16) $$\begin{array}{c} E\left[\log E\left(\zeta_{k};\lambda_{0}\right)\right]=-\frac{n_{z}}{2}E\left[\log\zeta_{k}\right]-\lambda_{0}E\left[\zeta_{k}\right], \end{array}$$

where $E\left[\log\zeta_{k}\right]=-\log\lambda_{0}-\gamma_{o}$, $\gamma_{o}$ is the Euler–Mascheroni constant and $E\left[\zeta_{k}\right]=\frac{1}{\lambda_{0}}$.

(A17) $$\begin{array}{c} E\left[\log N\left(x_{k};m_{k|k},P_{k|k}\right)\right]=-\frac{n_{x}}{2}\log\left(2\pi \right)-\frac{1}{2}\log\left(\det\left(P_{k|k}\right)\right)-\frac{n_{x}}{2}, \end{array}$$

where $n_{x}$ denotes the dimensions of measurement vector $x_{k}$.

(A18) $$\begin{array}{cc} E[\log\mathrm{GIG}\left(\zeta_{k};a_{k|k},b_{k|k},c_{k|k}\right)] & =\frac{c_{k|k}}{2}\log\left(\frac{a_{k|k}}{b_{k|k}}\right)-\log\left(2B_{c_{k|k}}\sqrt{a_{k|k}b_{k|k}}\right)\\ & \;+\left(c_{k|k}-1\right)E\left[\log\zeta_{k}\right]-\frac{1}{2}\left(a_{k|k}E\left[\zeta_{k}\right]+b_{k|k}E\left[\zeta_{k}^{-1}\right]\right), \end{array}$$

where

(A19) $$\begin{array}{cc} E[\zeta_{k}] & =\frac{\sqrt{b_{k|k}}B_{c_{k|k}+1}\left(\sqrt{a_{k|k}b_{k|k}}\right)}{\sqrt{a_{k|k}}B_{c_{k|k}}\left(\sqrt{a_{k|k}b_{k|k}}\right)}, \end{array}$$

(A20) $$\begin{array}{cc} E[\zeta_{k}^{-1}] & =\frac{\sqrt{a_{k|k}}B_{c_{k|k}+1}\left(\sqrt{a_{k|k}b_{k|k}}\right)}{\sqrt{b_{k|k}}B_{c_{k|k}}\left(\sqrt{a_{k|k}b_{k|k}}\right)}-\frac{2c_{k|k}}{b_{k|k}}, \end{array}$$

(A21) $$\begin{array}{cc} E[\log\zeta_{k}] & =\log\frac{\sqrt{b_{k|k}}}{\sqrt{a_{k|k}}}+\frac{\partial \log B_{c_{k|k}}\left(\sqrt{a_{k|k}b_{k|k}}\right)}{\partial \rho }{|}_{\rho =c_{k|k}}. \end{array}$$

(A22) $$\begin{array}{cc} E[\log\mathrm{IW}\left(R_{k};\Psi_{k|k},\nu_{k|k}\right)] & =-\frac{\nu_{k|k}}{2}\log\det\left(\Psi_{k|k}\right)-\frac{\nu_{k|k}+n_{z}+1}{2}E\left[\log\det\left(R_{k}\right)\right]\\ & \;-\frac{1}{2}\mathrm{tr}\left(\Psi_{k|k}E\left[R_{k}^{-1}\right]\right)-\frac{n_{z}\nu_{k|k}}{2}\log2\;-\log\Gamma_{n_{z}}\left(\frac{\nu_{k|k}}{2}\right). \end{array}$$

The expectations $E[\log\det\left(R_{k}\right)]$ and $E[R_{k}^{-1}]$ are, respectively, given by

(A23) $$\begin{array}{cc} E[\log\det\left(R_{k}\right)] & =\sum_{i=1}^{n_{z}}\psi \left(\frac{\nu_{k|k}+1-i}{2}\right)-n_{z}\log2+\log\det\left(\Psi_{k|k}\right), \end{array}$$

(A24) $$\begin{array}{cc} E[R_{k}^{-1}] & =\Psi_{k|k}^{-1}\left(\nu_{k|k}-n_{z}-1\right). \end{array}$$

Plugging (A9)–(A24) into (101) and performing some algebra, $L(q\left(x_{k}\right)q\left(R_{k}\right)q\left(\zeta_{k}\right))$ can be analytically computed.
